# Risk stratification in therapy-related myelodysplastic syndromes

**DOI:** 10.18632/oncotarget.21178

**Published:** 2017-09-24

**Authors:** Amer M. Zeidan

**Affiliations:** Section of Hematology, Department of Internal Medicine, Yale University, and Yale Comprehensive Cancer Center, New Haven, CT, USA

**Keywords:** myelodysplastic syndromes, therapy-related, prognostic tools

Risk stratification is crucial to the appropriate management of many cancers. However, in patients with myelodysplastic syndromes (MDS) for whom expected survival can vary from few months to decades and where the risk-adapted management strategies can vary from observation on one end all the way to immediate allogeneic hematopoietic stem transplantation (alloSCT) on the other end, it is quite apparent why accurate risk stratification is of paramount importance [1]. Therapy-related (t)-MDS, which makes up 10-20% of all cases, is descriptively diagnosed on the basis of prior exposure to chemotherapy and/or radiation therapy [2]. For a long time, t-MDS was considered a subset of MDS that is associated with associated with grave outcomes in all affected patients and therefore aggressive interventions including alloSCT were often recommended. In recent years, we started to appreciate that not all patients with t-MDS do very poorly [3]. In fact, some patients can live several years, which for a cancer with a median age at diagnosis of 76 years is quite substantial. How to best risk stratify patients with t-MDS remains unclear. While we have several well validated prognostic tools for *de novo* (d)-MDS, the datasets used for their development largely excluded patients with t-MDS. Furthermore, data regarding the performance of these scores in patients with t-MDS are scare and limited to small and/or single-center experiences.

In one of the largest cohorts to date, Zeidan and colleagues at the MDS Clinical Research Consortium analyzed the real-life outcomes of 370 patients with t-MDS, evaluated the prognostic utility of the risk stratification tools commonly used for d-MDS in this cohort, and compared the performance of these tools in the t-MDS cohort to a much larger cohort of 1950 patients with d-MDS [3]. The patients included in the t-MDS cohort resembled those commonly seen in practice; 76% were older than 60 years of age at diagnosis, and most had poor risk features such as having. 5% blasts in the bone marrow (BM, 43%) and poor risk karyotypes (49%). As expected, the overall survival (OS) of patients with t-MDS as a group was significantly worse than their counterparts with d-MDS (median, 19 vs 46 months, P<0.005). However, there was a substantial variation in OS between subsets of t-MDS as evidenced by the significantly different OS of the risk groups in each of the International Prognostic Scoring System (IPSS), revised IPSS (IPSS-R), MD Anderson Global Prognostic System (MPSS), WHO Classification-based Prognostic Scoring System (WPSS), and the MD Anderson t-MDS Prognostic Scoring System (TPSS). As an example, the median OS for t-MDS patients in the very low and the very high IPSS-R risk groups were 58 and 12 months, respectively [3].

Importantly, t-MDS patients within each risk group had a significantly inferior OS than d-MDS patients in the corresponding risk group of every prognostic tool. These observations suggested that variables not captured by these traditional clinicopathologic tools, such as genetic mutations and/or medical comorbidities, negatively impact the survival of patients with t-MDS. Using Akaike information criteria (AIC), a statistical measure of the relative fit goodness of models, the authors compared the relative prognostic utility of the 5 risk stratification tools in the t-MDS cohort and found that the MPSS, and to a lesser degree the TPSS and IPSS-R, provided the best discrimination of OS [3].

Prognostic tools are always limited by the fact that they separate patients into groups with significantly different survivals, but there could be significant variability in the survivals of patients within any one risk group. Incorporating the recurrent genetic mutations associated with independent prognostic impact into the traditional risk tools might be one way to improve the discriminatory performance and allow better prediction of survival for any individual patient. Such efforts are ongoing and it remains to be seen how these improved molecular-clinical tools would perform in the clinical setting, especially in the presence of logistical issues related to standardization of the performance and the interpretation of genetic panels in the community setting [4].

Another important consideration in the path towards development of improved risk stratification tools for t-MDS is how to define t-MDS using a biologic, rather than epidemiologic, fashion. Multiple studies suggested that many of the MDS cases diagnosed after prior chemotherapy or radiation might not be biologically and causally related and rather an incidental age-related occurrence [5–7]. Therefore, defining biologically-based ways of discriminating “real” t-MDS cases would be important for the development of a reliable prognostic tool for these patients [3, 8]. Creation of large databases that combine accurately collected genetic, clinical and laboratory/histologic data would be instrumental in these efforts. Not only would such databases potentially allow the creation of t-MDS-specific risk stratification tools, but they might uncover new alternations that could be targeted by rationally designed therapeutics or even allow the development of prevention strategies against the development of t-MDS in some patients who are at high risk following the delivery of chemotherapy and/or radiation for the primary malignancy.
